# Editorial: Recent advances in synthetic organic chemistry at the biomedical interface: honoring Professor Iwao Ojima on the occasion of his 80th birthday

**DOI:** 10.3389/fchem.2025.1721249

**Published:** 2025-10-16

**Authors:** Thierry Brigaud, Greta Varchi

**Affiliations:** 1 CY Cergy Paris Université, Centre National de la Recherche Scientifique (CNRS), BioCIS UMR 8076, Cergy-Pontoise, France; 2 Université Paris-Saclay, Centre National de la Recherche Scientifique (CNRS), BioCIS UMR 8076, Orsay, France; 3 Institute for the Organic Synthesis and Photoreactivity, Italian National Research Council, Bologna, Italy

**Keywords:** catalysis, green chemistry, anticancer drugs, antibacterial molecules, natural products, computational chemistry

This Research Topic brings together 20 contributions at the chemistry–biology–medicine interface honoring Professor Iwao Ojima's lifelong impact across organometallic catalysis, asymmetric synthesis, medicinal and natural products chemistry. The collection showcases how contemporary synthetic design, green chemistry, computation, and biology co-evolve to deliver selective probes, leads, and materials under milder and more sustainable conditions, while clarifying mechanisms relevant to cancer, infection, neurodegeneration, and immunology. In what follows, we outline the Topic's aims and contextualize the published articles in thematic frames, emphasizing convergences, and conclude with prospects that naturally flow from the body of work presented here.

A prominent trend across the Topic is the integration of greener synthesis with process intensification. For example, Pan and coworkers report a NiFe_2_O_4_@MCM-41@IL/Pt nanocatalyst that promotes A^3^ couplings to benzo [4,5]imidazo [1,2-a]pyrimidines under microwave irradiation in water. The system allows for simple magnetic separation and efficient reuse of the catalyst. This approach exemplifies how sustainability and reaction acceleration can work hand in hand to deliver pharmaceutically relevant heterocycles with reduced environmental impact (Pan et al.).

Methodological perspective also matters. A review on transition-metal strategies for the synthesis of anthracene scaffolds highlights how Pd/Ni cross-couplings, C–H activation, and ligands selection have expanded access to functional materials and therapeutic motifs, with a clear shift toward greener protocols (Sead et al.). In parallel, the Hosomi–Sakurai allylation’s enduring utility in total synthesis is recast through modern variants that deliver high yields, stereocontrol, and broad electrophile scope under mild conditions (Akwensi et al.). These elements underscore a recurring theme in the Research Topic: revisiting classic reactivity through contemporary catalysts and conditions is one of our fastest paths to better selectivity, lower waste, and more complex architectures.

Oncology runs like a spine through the Topic with height related contributions and showcases rational polypharmacology. Ether-type arylpiperazines were tailored into potent androgen receptor (AR) antagonists with SAR insights on cycloalkyl and ortho-methyl substitutions, supported by docking in the AR ligand-binding pocket (Jiang et al.). Hypoxia biology is addressed from two angles: a tail-engineered 4-pyridyl analogue of SLC-0111 selectively inhibits CA IX, induces G0/G1 arrest and apoptosis, and shows drug-like ADMET profile (Hashem et al.); a thiazole–chalcone/sulfonamide hybrid integrates tubulin inhibition with CA IX blockade, triggering intrinsic apoptosis with favorable selectivity (Khasawneh et al.). A Schiff-base Pd(II) complex outperforms cisplatin against DU-145 and PC-3 prostate cancer cell lines while being safer on fibroblasts, with docking suggesting dual engagement of the AR and apoptotic regulators (Pantic et al.). A quinolin-2(1H)-one series supplies dual EGFR/HER-2 inhibitors; lead 5a exceeds erlotinib, with kinase assays, apoptosis markers, and long-timescale MD supporting its mechanism of action (Al-Wahaibi et al., 2025). These results collectively argue that multi-target strategies, when anchored in mechanism, can counter redundancy and resistance without compromising selectivity. A novel series of thiazole-based derivatives was synthesized and tested for antiproliferative activity as dual EGFR/VEGFR-2 inhibitors. These compounds also presented antioxidant and antibacterial activities (Gomaa et al.). Phenylthiophosphoryl dichloride derivatives have been designed and evaluated for their antitumour and anti-inflammatory activities. The study included the evaluations of the H_2_S releasing capability of these compounds (Xu et al.). Novel pyrimidine-morpholine hybrids were designed and synthesized based on molecular hybridization approach. It was demonstrated that all derivatives had cytotoxic potential with IC_50_ in range of 5.12–117.04 μM. The biological activity of the compounds was confirmed by docking studies (Ataollahi et al.).

Anti-infectives and antipathogen approaches in the Research Topic emphasize antibiofilm action and complementary mechanisms. Efficiently built triazolyl heterocycles culminate in E10, which combines bactericidal activity with membrane disruption, strong antibiofilm performance, and notable anti-inflammatory effects. The same scaffold displays heavy-metal chelation capability, pointing to environmental remediation benefits and a resistance-resilient mode of action (Hong et al.). A second antibacterial approach introduces coumarin-tethered thiazoles that selectively hit problematic species and DNA gyrase, disrupt biofilms with low resistance risk, and show stable binding in docking and molecular dynamic studies (Ebaid et al.). In parasitology, thymol-anchored phenoxy-acetamides reduce oocyst burden *in vivo* and show encouraging ADME profile and docking to CpCDPK1, making 7 b a promising lead for further development (Rabee et al.).

Natural products inspired chemistry continue to expand our scaffold repertoire. From the desert endophyte *Phoma betae* comes phomaderide, a bis-spiro dimer with a rare 6/5/4/5/6 topology formed by stereoselective [2 + 2] photocycloaddition; its modest cytotoxicity and pronounced architectural novelty together make a strong case for analogue design and biosynthetic exploration. Building blocks–based molecular networking (BBMN) approach results as a modern path to targeted isolation from complex mixtures (Sun et al.). The Research Topic also probes phytochemical space functionally: LC-MS/MS-profiled *Capparis spinosa* extracts map specific phenolics to robust antioxidant performance and selective cytotoxicity in HCT-116 colon cancer cell line, and the authors show why orthogonal assays are essential to interpret antioxidant and anticancer potential rigorously (Oraibi et al.).

Computational approaches are not ancillary, representing a central theme of the Topic Research Topic, and a key tool for molecular design, prioritization of candidates, and mechanistic elucidation. Radiosensitization via Artemis inhibition is advanced through a pipeline that integrates cellular readouts, docking, ADMET, DFT, and 100-ns MD/MM-GBSA; compound 42 emerges with stable trajectories and favorable free energy, a template for converging *in silico* and *in vitro* evidence before medicinal chemistry expansion (Bashir et al.). In neurodegeneration, a repurposing-led analysis positions human neuraminidase as a tractable target for oseltamivir; docking, 50-ns MD, and enrichment of synaptic and kinase-related genes support the hypothesis while appropriately calling for experimental validation (Alzarea et al.). Across medicinal chemistry approaches elsewhere in the Topic, docking, MD, and DFT support SAR and target selectivity, evidence that computational layers are part of the prospective design loop rather than *post hoc* rationalization.

Translational determinants other than potency are also foregrounded as central considerations. A systematic review on flavonoids consolidates fragmented knowledge into comparative guidance: inclusion complexes and nanostructures boost AUC by ∼4.2× and ∼3.7× on average, while nanostructures and micelles can increase Cmax about 5.4×; the authors also highlight heterogeneity in solubility assays and urge standardization, a message that extends far beyond flavonoids (Taldaev et al.). In vaccine science, a streamlined route to the truncated linear trisaccharide of QS-21 removes a notorious synthetic bottleneck and opens the door to homogeneous adjuvant variants and clean SAR around a clinically important saponin (Lin et al.). Together with the green catalysis case studies above, these contributions remind us that exposure, supply, and manufacturability are decisive for translation.

Taken together, the 20 contributions span design-first medicinal chemistry, enabling synthesis, green catalysis, natural-product discovery, and computation-driven mechanism. They speak to a community increasingly comfortable blending reactivity innovation with systems-level biological thinking, all while embracing sustainability and scalability. As host editors, we are grateful to the authors and reviewers for assembling a Research Topic that both celebrates Professor Ojima’s legacy and points decisively toward the future. The next steps are clear: integrate data-driven design even earlier; hard-wire greener, recyclable workflows into standard practice; pursue multi-target and multi-modal strategies where biology demands it; and keep mining biodiversity and classical reactivity alike for scaffolds that medicine has not yet explored.

